# Venous Leg Ulcers: Advanced Therapies and New Technologies

**DOI:** 10.3390/biomedicines9111569

**Published:** 2021-10-29

**Authors:** Hubert Aleksandrowicz, Agnieszka Owczarczyk-Saczonek, Waldemar Placek

**Affiliations:** Department and Clinic of Dermatology, Sexually Transmitted Diseases and Clinical Immunology, University of Warmia and Mazury, Al. Wojska Polskiego 30, 10-959 Olsztyn, Poland; agnieszka.owczarczyk@uwm.edu.pl (A.O.-S.); w.placek@wp.pl (W.P.)

**Keywords:** venous leg ulcers, advanced therapies, new technologies, wound treatment, experimental dermatology

## Abstract

The prevalence of venous leg ulcers (VLUs) differs between 1.5% and 3% in the general population. The challenge in treating VLUs is common recurrence. Moreover, VLUs can be resistant to healing, despite appropriate treatment. In these cases, advanced wound therapies should be considered. The number of new technologies, applied in VLUs treatment, has increased in the last years. These therapies include biophysical interventions such as ultrasound therapy, electrical stimulations, electromagnetic therapy, or phototherapy. Furthermore, stem cell therapies, biologic skin equivalents, platelet-rich plasma therapy, oxygen therapies, anti-TNF therapy, or negative pressure wound therapy are advanced venous ulcer therapeutic methods that may support the standard of care. Medical devices, such as a muscle pump activator, or intermittent pneumatic compression device, may be especially useful for specific subgroups of patients suffering from VLUs. Some of the above-mentioned technologies require broader evidence of clinical efficacy and are still considered experimental therapies in dermatology.

## 1. Introduction

Chronic venous insufficiency (CVI) is a disease with numerous symptoms, including skin changes resulting from circulation disturbances caused by venous valve failure, venous reflux, an insufficient venous return, post-thrombotic syndrome, and venous hypertension [1,2,3,4,5]. Initially, venous leg insufficiency is clinically manifested by telangiectasias, varicose veins or interstitial edema. As the disease progresses, other symptoms such as hemosiderosis, lipodermatosclerosis, or stasis dermatitis on the legs can be observed [5]. Venous leg ulcer (VLU) is the most advanced form of this disease, often combined with searing pain, most often located on the inside surface of lower extremities between the ankle area and the mid-calf [1,5]. Moreover, untreated and long-lasting CVI can cause secondary lymphedema and the increased risk of cellulitis [5]. 

The risk factors for VLUs include advanced age, deep venous thrombosis, female gender, phlebitis, and obesity [2]. Owing to the increase in obesity and the aging population, the prevalence of chronic wounds, including VLU, is predicted to achieve epidemic proportions [6,7,8]. The disease has a negative impact on the quality of patients’ lives. Many of them live with chronic pain, experience issues with mobility, and perform everyday activities such as dressing or washing. The disease also has a significant impact on social life and mental health. About 30% of patients experience anxiety or depression [1,2]. 

VLUs are the most widespread type of leg ulcers. Their prevalence is rated between 1.5% and 3% in the general population and about 75% of leg ulcers have venous etiology [1,5]. 

Standard care in VLU treatment consists of local wound management and compressive therapy [9,10]. Correction techniques of pathological venous flow as a part of surgical and endovascular venous procedures are also included in the main therapeutic variants [9,11]. Patients’ education on self-care, which includes wound care, physical exercise, and diet, are an important part of treatment that leads to therapeutic success. Pharmacological, systemic treatment improving blood circulation, rheological properties of blood, and the local wound environment, such as pentoxifylline, sulodexide, or nutrition supplements are effective when linked with other standard wound treatment methods [10,12]. The gold standard of VLU treatment is currently the usage of a combination of methods mentioned above [13]. Conventional compression therapy is based on bandages or hosiery (stockings) with different degrees of pressure [9,10]. There are four aspects of wound bed preparation that should be systematically considered in order to allow the wound to heal. These aspects are: Tissue, inflammation/infection, moisture imbalance, and epithelial edge advancement, whose initials compose the TIME acronym [14] (Table 1).

Local wound management includes debridement, dressing techniques, and bacterial balance [10,12,15,16]. The occurrence of necrotic tissue detains the healing process of wounds. Debridement as the elimination of necrotic tissue from the area of a wound can reduce the healing time period. There are diverse methods of debridement, such as mechanical or enzymatic removal [15,17]. After debridement, other therapeutic methods can be applied, including skin transplants, in order to achieve wound closure [18]. The purpose of dressings in the treatment of wounds is to support healing processes and prevent wound infections. Dressings speed up fluid absorption, re-epithelialization, maintain moisture, and isolate thermal wounds. There are various types of dressings, such as moist occlusive dressings, hydrogels, or semi-permeable films [10,15]. Inflammation prolongs the healing process of wounds, which is why VLUs with clinical signs of infection should be treated with antibiotics according to the culture results. Depending on the severity of symptoms, systemic antibiotics or topical antimicrobials can be applied [10]. 

Despite appropriate treatment, the average time for healing VLUs varies from 6 to 12 months, and one-fifth of VLU cases do not heal within 24 months. If standard treatment fails, advanced therapy methods should be taken into consideration that can be applied as adjuvants to conventional treatment [19,20]. Another challenge in VLU treatment is frequent recurrence. Relapse after recovery within 5 years is high and reaches almost 70%. Furthermore, VLUs can be resistant to treatment. A lack of inclination to cure between 1.5 and 3 months or lack of healing within 12 months after optimal therapy is referred to as therapy resistance [1,21]. For these reasons, there is still an unmet need regarding insufficient treatment efficacy. It is thought that the weak healing of venous leg ulcers is caused by topical disturbances as a result of T lymphocytes and the amount of granulocytes increasing, oxygen deficiency, growth factor and cytokine imbalance [1]. Therefore, new therapies for chronic venous ulcers are still expected and the number of new wound management techniques increased in the last years and are sequentially being refined [1,12].

Key points about VLUs:

- Chronic venous insufficiency (CVI) in legs is a disease as a result of venous circulation disturbances;
- Venous leg ulcer (VLU) is the most advanced form of CVI;
- About 75% of leg ulcers have venous etiology which is the most common cause of chronic leg wounds;
- The risk factors for VLU include advanced age, deep venous thrombosis, female gender, phlebitis or obesity;
- Standard of care in VLU treatment consist of local wound management and compressive therapy;
- Despite appropriate treatment, the average time for venous leg ulcers (VLUs) healing varies from 6 to 12 months;
- VLUs can be resistant to treatment;
- 20% of VLUs cases do not cure within 24 months;
- In the case of VLUs standard treatment failure, advanced therapy should be considered.

## 2. Electrical Stimulations

Human cells produce endogenic electrical potentials around a wound which speed up the healing process by directing the migration of other cells to the site of a wound. Endogenous bioelectric fields (EBFs) participate in repair processes including chronic leg venous ulcers because they can influence cells and their gene expression, migration, proliferation, differentiation, or death. The epidermis layer disruption in the course of venous leg ulcers induces a constant voltage that harms cells, and also generates a flank electric field between 40 and 200 mV/mm. The fore-mentioned occurrence would potentially support faster ulcer repair throughout the galvanotaxis phenomenon. Galvanotaxis inhibitors, such as infectious or epigenetic agents, can disrupt an electric charge flow that localizes at the wound and inhibits regenerative processes. Various, exogenous forms of electrotherapy are used so as to stimulate regenerative processes in VLUs by imitating this physiological phenomenon [22,23]. 

Electrical stimulation (ES) induces angiogenesis by stimulating a signaling pathway of the mitogen-activated protein kinase (MAPK) and increasing the vascular endothelial growth factor (VEGF). Additionally, ES stimulates the signaling of VEGF receptors and therefore induces VEGF secretion and endothelial cells migration. Consequently, ES increases blood perfusion in the area of the ulcer. Furthermore, ES induces fibroblast proliferation by stimulation of the production of fibroblast growth factors (FGF). Additionally, ES increases the migration of fibroblasts and decreases the surface of chronic wounds. ES also reduces inflammation and regulates bacterial growth. Clinical studies have proved that ES reduces colony-forming units (CFU) of *S. aureus*, *P. aeruginosa*, and *E. coli* that generally colonize chronic ulcers. It is thought that the delivery of electricity modifies the pH of the bacteria’s surroundings which injures their cell wall, leading to an inflow of solutes and death [22]. 

We distinguish the following types of exogenous ES, such as direct current (DC), alternating current (AC), pulsed current (PC), and degenerate wave (DW). Unfortunately, nowadays it is not possible to indicate the most efficient sort of ES owing to variability in the study protocols and lack of comparable, head-to-head clinical studies. Nevertheless, PC in contrast to AC, has polarity, can better imitate the physiological current, and penetrate deeply into the skin [22,23]. 

The ES device is usually applied by setting electrodes around the ulcer and is generally painless, but patients can also report paresthesia or a tingling sensation around the area of electrode plates. There are also other ways of ES administration, such as bioelectric dressings, or wireless ES application applied on the ulcer. The ES parameters can be regularized to wave amplitude, pulse type, different frequencies, and exposure duration. Current scientific evidence confirms the possibility of using ES as an adjuvant therapy in chronic leg ulcers treatment. Nonetheless, there is still a need to conduct trials assessing an optimal application method, recommended ES parameters, or exposure duration. It is considered that electro-active biomaterials or wearable, self-sustainable ES can act as wound care management in the future [22].

## 3. Ultrasound Therapy

Ultrasound therapy (UT) is pointed as one of the adjunctive treatments used in venous leg ulcers therapy [20,24]. A mechanical effect is the major result of ultrasound use [20]. There are two types of ultrasound influence on tissues: thermal and non-thermal. The non-thermal action of ultrasound is thought to be due to two phenomena: acoustic streaming and cavitation. Acoustic streaming means confluence and movement of particles located inside a fluid medium caused by the physical strength of sound waves. We differentiate two sorts of this streaming: microstreaming, mechanically stronger and bulk streaming [24]. The cavitation makes and sets in motion micron-sized bubbles in a fluid medium caused by sound waves [20,24]. Stronger application of ultrasound can cause a tissue temperature increase to around 40 degrees Celsius. This thermal effect induces blood flow rise in tissue and beneficial, physical modifications in collagen structures. It is also reported that ultrasound induces protein synthesis, cell proliferation, angiogenesis, or enzymatic fibrinolysis. Moreover, ultrasound induces fibroblasts for collagen formation, enhances collagen deposition, and speeds up granulation tissue creation. Additionally, ultrasound has an anti-inflammatory effect, reduces tissue edema, and supports the cleansing of necrotic tissue from the wound [20,24]. On the other hand, the influence of ultrasound waves on healing tissues is not completely known, and its effects on the healing process in vivo is not clear [20]. 

There are two types of therapeutic ultrasound, either high-frequency ultrasound (HFU) (1–3 MHz) or low-frequency ultrasound (LFU) expressed in the kilohertz (30–40 kHz), and both kinds are applied in the treatment of VLUs [20,24]. Ultrasound devices can supply a variety of frequencies in a pulsed, or constant way, and are usually administered for between five and ten minutes each time. Ultrasound can be applied directly to the skin, more often around the ulceration than to the bottom of the ulcer. A coupling agent is usually placed between the applicator head of an ultrasound device and the skin surface [24]. Indirectly applied ultrasound is administered through a water bath or saline mist placed on a wound bed by using the non-contact ultrasound device [20,24]. Crucial factors in the administration of ultrasound are the ultrasound applicator head motion, selection of the exposure time, and intensity of ultrasound wave [24]. 

According to the results of several clinical trials, administration of low dose ultrasound shows greater efficiency in the healing of skin wounds than high dose ultrasound application [20]. Furthermore, HFU can entail more side effects, such as skin burns, pain, or endothelial injury [20,24]. UT can accelerate a decrease in venous leg ulcer surface, reduce associated pain, and the average duration of the ulcers [20].

Unfortunately, based on a review of ultrasound randomized controlled trials most of the evidence is of poor quality and it is not clear whether therapeutic UT accomplishes the healing process of venous leg ulcers [24].

## 4. Electromagnetic Therapy

Electromagnetic therapy (EMT) is used as an additional treatment supporting wound healing including venous leg ulcers. Other names of EMT that can be found in the literature are bioelectricity, electromagnetism, magnetic field therapy, magnetic healing, or magneto biology. Interest in its application in wound treatment has increased over the recent years. 

EMT utilizes electromagnetic energy, and EMT devices produce a field effect. There are two kinds of electromagnetic fields induced by EMT devices: pulsed electromagnetic field (PEMF) and continuous electromagnetic field. The pulsed electromagnetic field, as opposed to continuous electromagnetic field, induces a brief continuance of the pulses. This beneficial phenomenon can protect the tissues against a potential injury caused by heat produced by continuous electromagnetic field devices. Unfortunately, there is no standardization of PEMF devices usage including exposure duration, length of exposure intensity, type, or frequency of the electromagnetic field.

It is hypothesized that PEMF may generate an electric signal on the damaged cell membrane. This commences a series of physiological actions supporting wound healing, such as a wander of electrically loaded cells engaged in wound repair. Therefore, the quantity of fibroblasts and macrophages increases in the wound. There is data that suggest that PEMF also influences the generation of free radicals inside cells, which mediates intracellular liaison. Moreover, PEMF increases fibrin and collagen deposition and reduces inflammation. Consequently, tissue recovery and cell proliferation in wounds can be observed. 

The results of clinical trials on EMT usage for the treatment of venous leg ulcers have inconsistent results. Some of them confirm the accelerated healing process of venous leg ulcers in the form of reduction in the dimension of the wound. Others, in turn, report no such effect of EMT on the treatment process. Similar observations concern the effect of EMS on the reduction of pain associated with venous leg ulcers. There are studies that confirm the beneficial effects of EMT on pain, others have not proved so. Adverse reactions reported in clinical trials with EMT included: sensations of tingling or heat in the low extremities, and headaches.

Due to the weak quality of electromagnetic therapy evidence, the clinical effect of ETM on the venous leg ulcers healing process is unclear [9].

## 5. Photobiomodulation with Low-Level Light Therapy

Photobiomodulation (PBM) or low-level light therapy (LLLT), as a variant of phototherapy, is another, promising venous leg ulcer healing technology [11,25]. The light generated with energies lower than 10 J/cm^2^ or power of lower than 1 watt is defined as LLLT. Nowadays, either lasers or light-emitting diodes (LEDs) are categorized as kinds of LLLT [25]. LEDs can induce red light, blue light, or near-infrared wavelengths, generate energy as photons, and do not enhance the light energy like lasers [19,25]. Lasers produce energy by stimulated emission that can be applied in several modes, such as continuous, pulse, or alternating. Laser devices include an active medium that co-operates together with produced electromagnetic waves to intensify the light. The active medium of lasers may consist of gas, liquid, solid, or semiconductor. 

Photobiomodulation means cell activation through the photo-chemical effect achieved by LLLT devices usage. This effect is based on the cellular absorption of energy from the light which induces their chemical, and physical transformations without heat generation [13,25].

It is considered that light energy is absorbed by molecule photoreceptors called complex IV localized on the respiratory chain inside mitochondria. The fore-mentioned enzymatic modification begins biochemical pathways and supports Na^+^/K^+^-ATPase activity. Consequently, it props the process into chemical energy and increases ATP production [13,25]. Moreover, LLLT releases serotonin, bradykinin, and histamine which also induce ATP production and restrain prostaglandins production [13]. Consequently, it leads to cell activity enhancement and restores its homeostasis [13,25]. Furthermore, LLLT inflects cell function by nitric oxide modulation. This cellular activity accelerates tissue repair, granulation tissue formation, cell proliferation, pain reduction, anti-inflammatory modulation, bacterial balance, and synthesis of proteins [13,19].

Generally, LLLT in the treatment of venous leg ulcers is a contactless method, and LLLT devices usually direct the light beam around the whole wound surface [25]. A new LLLT method is a photo-converter wound gel applied to the venous ulcers and illuminated by an LED lamp. This gel includes light-absorbing particles but is not absorbed by skin cells. The chromophores located in the gel, upon LED illumination, emit micro-pulsed photons as fluorescence in the form of the visible light spectrum [19].

Low-level laser therapy may help in the treatment of venous leg ulcers as the adjuvant alternative, but stronger evidence is required [13,25]. A high-quality systematic review is expected to confirm that [25].

## 6. Oxygen Therapy

Several healing processes occurring in tissues such as cell replication, anti-bacterial macrophages act, necrotic tissue removal, or collagen formation are greatly oxygen relative. 

A commonly known fact is that chronic wound tissues have a too low amount of oxygen, and the healing process of hypoxic tissues is disturbed, especially when a transcutaneous oxygen partial pressure (pO2) is lower than 40 mmHg [14,26]. It is suggested that oxygen therapy (OT) increases oxygen delivery to wounds, accelerates their healing, and does not reveal relevant cell damage risk [14,27,28].

### 6.1. Hyperbaric Oxygen Therapy

Hyperbaric oxygen therapy (HBOT) is used to increase the oxygen supply to wounds and involves patients breathing with pure (100%) oxygen under increased pressure inside a special compression chamber [14]. The whole treatment cycle usually consists of 15 to 40 therapeutic procedures lasting from one hour to two hours each [14,27]. These procedures happen in pressure conditions from 2.0 to 2.5 atmospheres absolute (ATA) and are held once or twice a day [14,29] and lead to increased oxygen accumulation to a wound. 

As the beneficial results of hyperbaric oxygen therapy are noted: fibroblast activation, antibacterial effect, increase in growth factors, tissue hyper-oxygenation, decrease in inflammatory cytokines, leukocyte chemotaxis reduction, angiogenesis, increased tissue perfusion, and oedema reduction [14,27].

Clinical implications under the influence of hyperbaric oxygen therapy, noted in trials, are reduction in the size of the venous leg ulcers, and acceleration of the healing process [14,27,28,29]. Nevertheless, there is still a need to conduct high-quality, large randomized, future trials to evidence whether HBOT as an adjunct treatment for non-healing venous leg ulcers improves a long-term disease state [14].

### 6.2. Topical Oxygen Treatment

Topical oxygen therapy (TOT) is an innovative technology in the management of resistant-to-healing venous leg ulcers. TOT is applied directly to the wound and does not require a full-body compression chamber. That is why TOT has much lower systemic complications and can be the alternative for patients with contraindication to HBOT [26,30].

Generally, TOT is used once a day for 90 min and supplies pure (100%) oxygen at a pressure of just over 1 atmosphere. New therapeutic methods of TOT supply oxygen continuously for up to 72 h [26,30]. 

According to the literature, TOT speeds up wound healing by accelerating epithelialization, MRSA elimination, stimulation of circulation, edema reduction, vascular endothelial growth factor (VEGF) level increase, and stimulation of granulation in the wound [26,30]. Nevertheless, most literature data on the TOT mechanism of action refers to HBOT, thus has limited value [26].

Clinical trials confirmed that TOT could influence venous leg ulcers area reduction, pain relief, or decrease ulcer recurrence rate [26,28,30]. On the other hand, the poor quality of current evidence requires clinical confirmation in the future. Some clinical data suggest that topical oxygen diffuse is too superficial and the oxygen amount absorbed by the wound is insufficient. That is why there are authors who claim that TOT should not be applied nowadays beyond clinical trials [26].

## 7. Negative Pressure Wound Therapy 

Negative pressure wound therapy (NPWT) is a technology supporting the standard of care in VLUs. NPWT accelerates the healing process of venous leg ulcers with several mechanisms such as local edema reduction, reduction in bacteria, inflammatory mediators, and wound exudate. Moreover, NPWT provides angiogenesis induction, promotes tissue perfusion, stimulates tissue granulation, causes wound shrinking, and contraction of its edges. These mechanisms improve topical wound state and facilitate the healing process [11,18,31,32]. Other names for this therapy found in the literature are: vacuum-assisted closure (VAC), vacuum sealing technique (VST), vacuum pack therapy, sealed surface wound suction (SSS), subatmospheric pressure dressing (SPD), foam suction dressing, or sealing aspirative therapy [33].

The device for negative pressure therapy generates subatmospheric pressure and consists of a special pump linked to a container collecting wound exudation. An airtight dressing surrounds the wound and its exudate is drained into the container through a pipe. The dressing applied to the wounds is usually changed every 48–72 h, and the treatment duration is at least several weeks [32,33]. Due to the specificity of VAC treatment, compression therapy as the primary treatment of VLUs is not applied in parallel. Furthermore, the size of conventional NPWT devices means that this therapy takes place primarily in hospital settings. For these reasons, this technology is not widely used in the treatment of VLUs. A new alternative for negative pressure therapy is ultraportable devices. Their significant innovation is the lack of a container whose function is taken over by absorbent dressings linked to a suction pump that can be placed in the bandage. These ultraportable devices, due to their smaller dimensions than the conventional NPWT devices, do not restrict patients’ mobility. The advantage of this new technology is also the possibility of parallel use of compression therapy [11].

A few clinical trials conducted in patients with VLUs confirmed the effectiveness of NPWT in the field of wound surface reduction. Nevertheless, owing to poor evidence, there is still a need to conduct large, strict, randomized trials to confirm such efficacy for VLUs patients [18,32].

## 8. Platelet-Rich Plasma Therapy

The wound repair process continues as a result of the interplay of the intercellular matrix, skin cells, and plasma proteins [34]. One type of these proteins, also circulating in the blood, are growth factors (GFs). GFs affect the genes of wound cells, thus modulating the regeneration processes, such as proliferation, differentiation, or migration. Consequently, GFs induce extracellular matrix synthesis and angiogenesis. The inadequate GFs bioavailability is characteristic of chronic wounds. Therefore, the local delivery of growth factors has been identified as a promising therapeutic option [35]. Activated platelets release GFs from their α-granules, and together with other plasma proteins, such as fibronectin or fibrin, are significant agents in regenerative processes [36,37]. Platelets as a natural source of growth factors have been recognized as an excellent material for tissue regeneration [37].

Platelet-rich plasma (PRP) or autologous platelet-rich plasma is platelet suspension extracted from whole blood [36,37]. The concentration of platelets in PRP is higher from two to six times than that occurring in blood [35]. To form a liquid or a gel containing multiple growth factors, PRP is most often mixed with thrombin. PRP supplies not only numerous growth factors but also signaling cytokines that also play a crucial role in new tissue synthesis, angiogenesis, or regulation of inflammation [36]. PRP is used in various fields of medicine including chronic wound treatment and is applied in a form of injections or topically in a form of gel on a weekly basis for at least several weeks or months [34,35,36].

Published studies in the treatment of VLUs have revealed the effectiveness of PRP therapy in reducing the area of the ulcers, and an improvement in patients’ quality of life [34,36,37]. Moreover, the antibacterial effect of PRP in wound treatment has been shown. Nonetheless, there are no standardized methods for obtaining PRP, and larger, well-designed clinical trials in this field are needed [36,37]. That is why autologous PRP therapy is nowadays an alternative treatment method for VLUs, and as a biocompatible procedure, it is considered safe [34,36].

## 9. Biologics

Biologics are mainly monoclonal antibodies that target specific parts of the immune system by blocking proteins or cell receptors. Proteins in the immune system, such as tumor necrosis factor-α (TNF-α), interleukins (ILs) 12 and 23, or interleukin (IL) 17 play a crucial role in the pathogenesis of many diseases such as psoriasis, psoriatic arthritis, rheumatoid arthritis, or ankylosing spondylitis. Studies have demonstrated the importance of TNF-α in the development of VLUs, and IL12, IL23, or IL17 are considered as potential therapeutic targets for the treatment of wounds including VLUs [38,39,40,41,42,43].

Non-healing wounds have increased pro-inflammatory cytokine accumulation, and persistent inflammation in chronic wounds hinders the healing process. Elevated cytokine amounts adversely influence the homeostasis of cells necessary for proper collagen production [38,40]. Non-healing wounds have also been shown to have significantly higher levels of TNF-α than healing wounds [39]. It has been proven that the duration of VLU is correlated with the level of TNF-α, and the level of which increases the longer the ulcer is sustained [38]. It has been also proven that TNF-α levels increase in VLUs, either systemically or locally [38,40]. Anti-TNF-α agents, such as adalimumab, etanercept, or infliximab reduce inflammatory cytokine levels, TNF-α binding, and fibroblast apoptosis. Moreover, biologics decrease circulating leukocytes, and increase wound collagen. A pilot study has been performed with adalimumab administrated subcutaneously and has confirmed the potential efficacy of this therapy in the treatment of VLUs by wound size reduction [38]. There are also considerations to verify the effectiveness of anti-TNF-α therapy in direct application to chronic wounds [41]. It has been proven that direct appliance of etanercept to chronic wounds may diminish the inflammatory action of TNF-α, and therefore the reduction in the cytotoxic impact of the wound fluid on fibroblasts. It may consequently improve wound healing [40,41].

There are considerations to verify the efficacy of other pro-inflammatory cytokine inhibitors in chronic wound therapy, such as inhibitors of IL12, IL23, or IL17 [42,43]. The IL-17 family consists of a collection of pro-inflammatory cytokines, such as IL-17A, IL-17B, IL-17C, IL-17D, IL-17E/IL-25, and IL-17F that are involved in the pathogenesis of many immune-mediated diseases. The IL-17 family is also considered that it may play a crucial role in the pathogenesis of chronic wounds including VLUs. Nevertheless, studies with IL17 inhibitors have not yet commenced in this indication [42]. Similarly, the efficacy of IL 12 and IL23 inhibitors in the treatment of VLUs requires clinical confirmation [43].

Biologics were a breakthrough in the treatment of many auto-inflammatory diseases, such as psoriasis, psoriatic arthritis, rheumatoid arthritis, or ankylosing spondylitis [38,39,42]. If future trials confirm the clinical efficacy of biologics in the treatment of VLUs, this therapy may significantly accelerate the wound healing process.

## 10. Stem Cell Therapy

Stem cells are undifferentiated cells that have the capability to self-renew and are able to differentiate into various cell types [15,44,45]. There are two types of stem cells: embryonic stem cells and adult stem cells. Embryonic stem cells are insulated from blastocysts, whereas adult stem cells are located in most tissues [44]. Stem cells from various sources show a large potential for wound healing acceleration and can be used for wound repair. Stem cells play a significant role in each stage of the wound healing process, and their dysfunction leads to chronic wound development [15,45]. Stem cells release growth factors, correct weakened signaling growth factor pathways, and provide significant cytokines and chemokines. These cells, through paracrine influence, hasten the wound healing process. Moreover, stem cells modify the inflammatory process, accelerate angiognenesis, and re-epithelialization, differentiate into myofibroblasts. Therefore, they increase wound constriction, collagen deposition, and enhance wound closure [44,45]. Furthermore, these cells also reduce scarring [45]. The major sources of adult stem cells that can be applied for wound reparation are: adipose tissue, bone marrow, and blood. Adipose stem cells can be insulated from adipose tissue by liposuction procedure. Mesenchymal stem cells isolated from the bone marrow are mainly taken from the femur iliac crest. There are other sources of stem cells that can also be used for wound healing, such as keratinocyte stem cells, epidermally-derived mesenchymal stem cells, fibroblast stem cells, induced pluripotent stem cells, placental mesenchymal stem cells, and umbilical cord mesenchymal stem cells [44,45]. There are autologous or allogeneic stem cell therapies depending on whether the stem cell donor and recipient are the same person or not [15,44,45,46]. Among many types of adult stem cell, adipose-derived stem cells, and mesenchymal stem cells are assumed to be the most appropriate candidates for increasing tissue regeneration. Recently, adipose-derived stem cells have become more popular than bone marrow-derived stem cells owing to the easier possibility of obtaining from tissues [46,47].

Clinical data has revealed that stem cell therapy (SCT) fostered the healing process in each wound repair phase. Clinical trials proved the improvement of chronic venous ulcer healing as a result of the SCT use, with a crucial decrease of wound surface and high-quality tissue regeneration [15,44,45,46,47]. SCT is a novel treatment method for VLUs and future trials will determine what significance that option will have. There is insufficient data on long-term results of skin wound treatment using such therapy and broader studies are required. Moreover, the determination of the perfect source of stem cells and the improvement of stem cell supply methods are needed for wider clinical application of this option in VLUs treatment [15,44,45,46,47].

## 11. Other Advanced Therapies and New Technologies

Other advanced therapies and new technologies can influence the individual aspects of the TIME wound bed preparation (Table 2). There are also other advanced wound-healing technologies that support of venous return, such as muscle pump activator (MPA) or intermittent pneumatic compression (IPC) [2,5].

### 11.1. Muscle Pump Activator (MPA)

Muscle pump activator (MPA) device works by neuromuscular electrostimulation (NMES) and stimulates the common peroneal nerve which causes calf muscles isometric activation. Consequently, a stimulated calf muscle pump induces venous return enhancement, and causes edema and venous stasis reduction [2]. This therapy may be particularly advantageous in patients with limited mobility.

### 11.2. Intermittent Pneumatic Compression (IPC)

Intermittent pneumatic compression (IPC) devices are effective in the process of healing venous ulcers for patients with lymphedema. The advantage of this therapy is the possibility of self-application in contrast to the traditional compression therapy that requires application by a third party. According to clinical trials results, IPC is not more efficient than standard compression [5].

Other therapies categorized as new technologies in VLU treatment that may facilitate wound healing are: biologic skin equivalents, such as bilayered living cellular construct (BLCC), or 3D-printed hydrogel dressing [8,13].

## 12. Conclusions

Venous leg ulcers are still a huge therapeutic challenge. Despite the continuous progress and the development of new therapeutic technologies, the rate of leg wound healing is still unacceptable for many patients. The biotechnology and pharmaceutical industries have been constantly working on the improvement of existing therapies and the development of new wound-healing technologies to promote therapy effectiveness and increase the quality of patients’ lives. However, most of the advanced therapies and new technologies for the treatment of VLUs do not have strong scientific evidence or are in the initial stages of determining their therapeutic usefulness. New therapeutic options of VLU treatment still require large, high-quality, randomized clinical trials to confirm their clinical suitability [9,12,13,24,25]

## Figures and Tables

**Table 1:** The TIME concept summary.

TIME Acronym	Aspect to Access	Type of Intervention to Promote Wound Healing
T	Tissue	Wound debridement from all the following: exudate, necrotic tissue, slough, biofilm, or foreign bodies, if present
I	Inflammation/Infection (assessment of the wound for potential infection and inflammation)	Topical antimicrobials and/or systemic antibiotics in case of infection
M	Moisture imbalance (assessment and fluid/exudate wound management)	Dressing
E	Epithelial edge advancement	Debridement, acellular dermal matrices, skin grafting, or adjunctive therapies in case of dry or macerated wound edges

**Table 2:** The influence of the VLUs advanced therapies/new technologies on the individual aspects of the TIME wound bed preparation.

TIME Acronym	Advanced Therapy
T	Negative pressure wound therapy (NPWT) Electrical stimulations (ES) Ultrasound therapy (UT) Electromagnetic therapy (EMT)
I	Electrical stimulations (ES) Ultrasound therapy (UT) Electromagnetic therapy (EMT) Low-level light therapy (LLLT) Oxygen therapy (OT) Platelet-rich plasma (PRP) Stem-cell therapy (SCT) Biologics
M	3D-printed hydrogel dressing
E	Electrical stimulations (ES) Ultrasound therapy (UT) Electromagnetic therapy (EMT) Bilayered living cellular construct (BLCC) Oxygen therapy (OT) Low-level light therapy (LLLT)

## Data Availability

Not applicable.
